# Biosynthesis and molecular actions of specialized 1,4-naphthoquinone natural products produced by horticultural plants

**DOI:** 10.1038/hortres.2016.46

**Published:** 2016-09-21

**Authors:** Joshua R Widhalm, David Rhodes

**Affiliations:** 1 Department of Horticulture and Landscape Architecture, Purdue University, 625 Agriculture Mall Drive, West Lafayette, IN 47907-2010, USA

## Abstract

The 1,4-naphthoquinones (1,4-NQs) are a diverse group of natural products found in every kingdom of life. Plants, including many horticultural species, collectively synthesize hundreds of specialized 1,4-NQs with ecological roles in plant–plant (allelopathy), plant–insect and plant–microbe interactions. Numerous horticultural plants producing 1,4-NQs have also served as sources of traditional medicines for hundreds of years. As a result, horticultural species have been at the forefront of many basic studies conducted to understand the metabolism and function of specialized plant 1,4-NQs. Several 1,4-NQ natural products derived from horticultural plants have also emerged as promising scaffolds for developing new drugs. In this review, the current understanding of the core metabolic pathways leading to plant 1,4-NQs is provided with additional emphasis on downstream natural products originating from horticultural species. An overview on the biochemical mechanisms of action, both from an ecological and pharmacological perspective, of 1,4-NQs derived from horticultural plants is also provided. In addition, future directions for improving basic knowledge about plant 1,4-NQ metabolism are discussed.

## Introduction

**Figure 1:**
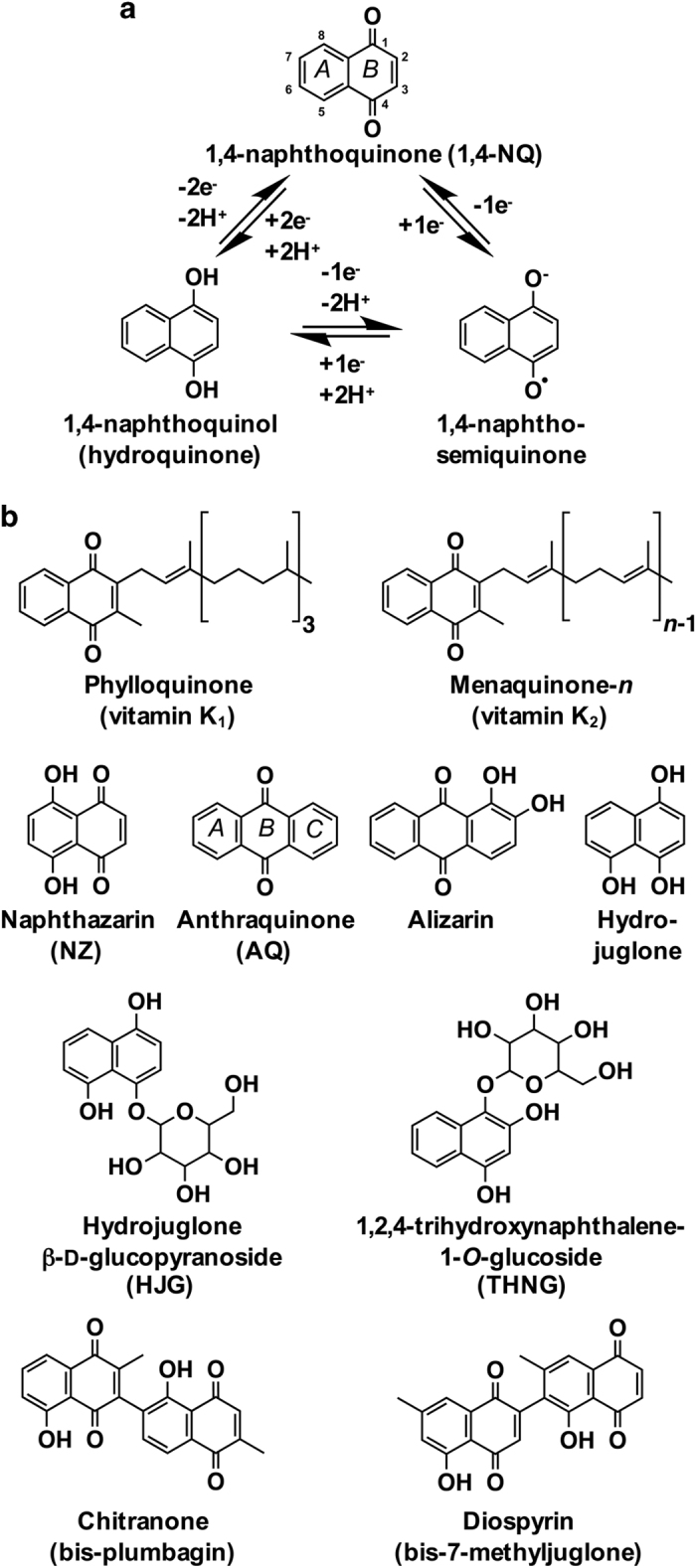
(**a**) Basic structure and redox forms of 1,4-NQs and (**b**) examples of 1,4-NQ natural products referenced in the text.

The 1,4-naphthoquinones (1,4-NQs) are redox active compounds structurally related to naphthalene that are comprised of a benzene moiety (ring *A*) linearly fused with a fully conjugated cyclic diketone (ring *B*) in which the carbonyl groups are arranged in the *para* orientation (Figure 1a). In living organisms, 1,4-NQs encompass a class of natural products containing a 1,4-naphthalenoid ring, often bearing one or more methyl, hydroxyl and/or methoxy substitutions, and, in some molecules, a liposoluble side chain.

The 1,4-NQs are synthesized by organisms throughout all kingdoms of life (described below) and are involved in vital metabolic processes and/or contribute toward adaptation to ecological niches. Filamentous fungi synthesize dozens of 1,4-NQ-based compounds,^1^ some of which are reported to be responsible for coloring of sexual fruiting bodies and thought to confer protection against ultraviolet, desiccation and insects.^2^ Although restricted to only a handful of lineages, several animals also produce 1,4-NQs, such as those found in secretions of a few tenebrionid beetles^3^ and in the scent-producing glands of certain arachnids.^4^ Moreover, the sea urchin, *Strongylocentrotus purpuratus*, is reported to make a red-colored 1,4-NQ called echinochrome in its pigment-producing cells.^5,6^ Within bacteria, the *Actinomycetes* produce numerous 1,4-NQs,^7^ as well as substituted 5,8-dihydroxy-1,4-NQs called naphthazarins (NZs; Figure 1b) that form core moieties in the antimicrobial rubromycins.^8^ Many extant archaea and bacteria have retained the ability to synthesize menaquinone (vitamin K_2_; Figure 1b), a prenylated 1,4-NQ suggested to be the ancestral quinone involved in anaerobic respiratory electron transport chains.^9^ In some cyanobacteria, rhodophytes (red algae)^10^ and most diatoms (protists),^11^ menaquinone fulfills the role of phylloquinone (vitamin K_1_; Figure 1b), which is the 1,4-NQ involved in photosynthesis in plants,^12^ green algae,^13^ many cyanobacteria^9^ and some euglenoids (for example, *Euglena gracilis*^14^).

Perhaps the greatest diversity of 1,4-NQs is found amongst the specialized natural products synthesized by plants, particularly those by horticultural species (see refs 7,15,16,17,18,19,20 for further information on the occurrence of plant 1,4-NQs). Collectively, using several different metabolic pathways, plants produce hundreds of specialized 1,4-NQs, NZs and derived metabolites, including certain anthraquinones (AQs; Figure 1b). Together, these natural products possess a multitude of biochemical properties modulating numerous ecological and pharmacological roles, offering new targets for addressing challenges in modern horticulture and providing scaffolds for developing novel drugs.

This review summarizes the current knowledge on the different plant biosynthetic pathways involved in forming simple 1,4-naphthalenoid rings and on the metabolism of downstream 1,4-NQs derived from horticultural species. Advances made in uncovering the molecular mechanisms of action, ecological functions and pharmacological activities of select specialized horticultural plant 1,4-NQs are also highlighted. Table 1 summarizes the horticultural species and 1,4-NQ natural products covered in this review. As the production of some 1,4-NQ natural products involves intermediates shared in phylloquinone biosynthesis, relevant discoveries that have improved the understanding of this primary metabolic pathway in *Arabidopsis thaliana* will also be described. However, more comprehensive reviews on this pathway have recently become available,^21,22^ as have reviews concerning the metabolism of precursors for each of the 1,4-NQ biosynthetic pathways (for example, for the shikimate pathway,^23^ benzoic acids,^24^ isoprenoids^25^ and polyketides^26^). Finally, this report will cover future directions for addressing gaps still remaining in understanding specialized plant 1,4-NQ metabolism.

## Plants have evolved several pathways to synthesize 1,4-naphthalenoid rings

**Figure 2:**
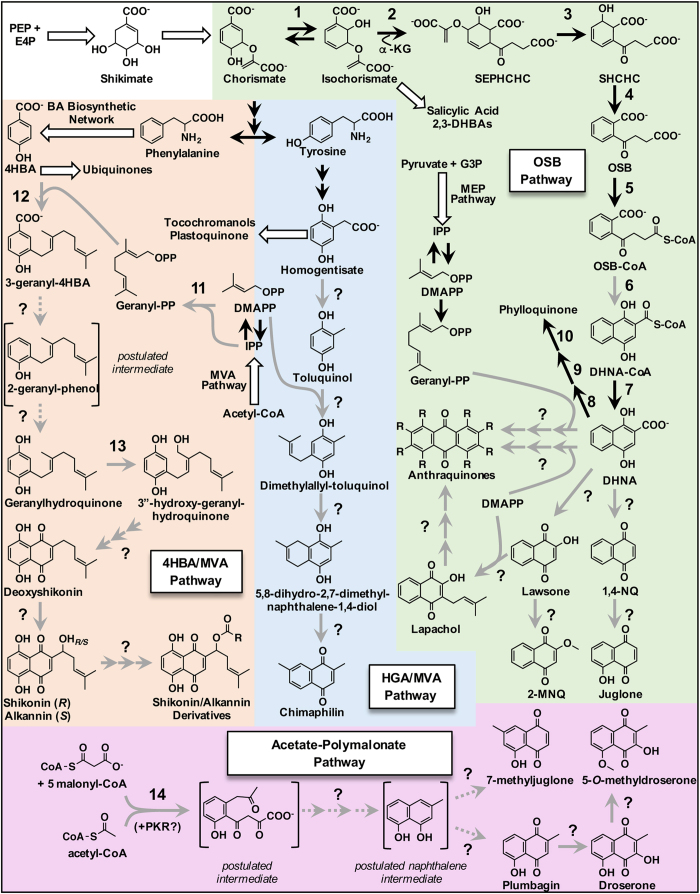
The plant 1,4-NQ biosynthetic network. Presented is the current understanding of the enzymes and intermediates involved in the core metabolic pathways for synthesizing 1,4-naphthalenoid rings in plants and for producing some of the major horticultural 1,4-NQs. Subcellular architecture is not depicted but is discussed in the text. Black arrows indicate the existence of genetic evidence to support biosynthetic reactions, while gray arrows signify a lack of genetic evidence. Tandem triplicate arrows indicate an unknown number of multiple steps to go from a given intermediate to the next metabolite. Dotted arrows are used to represent steps to and from postulated intermediates. White block arrows represent entire metabolic pathways to relevant or noteworthy metabolites not addressed in this review. Question marks next to arrows indicate that enzymatic activities for those steps have not been described. Numbers next to arrows represent characterized enzymes or detected enzymatic activities: 1, isochorismate synthase; 2, 2-succinyl-5-enolpyruvyl-6-hydroxy-3-cyclohexene-2-carboxylate (SEPHCHC) synthase; 3, 2-succinyl-6-hydroxy-2,4-cyclohexadiene-2-carboxylate (SHCHC) synthase; 4, *o*-succinylbenzoate (OSB) synthase; 5, OSB-CoA ligase; 6, Dihydroxynaphthoyl-CoA (DHNA-CoA) synthase; 7, DHNA-CoA thioesterase; 8, Dihydroxynaphthoic acid (DHNA) phytyl transferase; 9, NAD(P)H dehydrogenase C1 (NDC1); 10, Demethylphylloquinone methyltransferase; 11, cytosolic geranyl diphosphate synthase (GPPS); 12, *p*-hydroxybenzoate:geranyltransferase (PGT); 13, geranylhydroquinone (GHQ) 3″-hydroxylase; 14, polyketide synthase (PKS). BA, benzoic acid; DHBA, dihydroxybenzoic acid; DMAPP; dimethylallyl diphosphate; E4P, D-erythrose 4-phosphate; G3P, glyceraldehyde 3-phosphate; IPP, isopentenyl diphosphate; MEP, methylerythritol 4-phosphate; MVA, mevalonic acid; PEP, phosphoenolpyruvate; PKR, polyketide reductase; PP, diphosphate.

In nature, 1,4-NQs are known to be derived from several metabolic pathways: the *o*-succinylbenzoate (OSB; Figure 2) pathway; the 4-hydroxybenzoic acid (4HBA; Figure 2)/geranyl diphosphate (GPP; Figure 2) pathway; the acetate-polymalonate pathway; the homogentisate (HGA; Figure 2)/mevalonic acid (MVA) pathway; and the futalosine pathway. Except for the futalosine pathway, which was recently discovered to be an alternative route toward menaquinone in some bacteria,^27^ all of these pathways are present in the plant kingdom. In the OSB, 4HBA/MVA and HGA/MVA pathways, chorismate, the product of the shikimate pathway,^23^ ultimately provides one of the rings in the core 1,4-naphthalenoid structure, although the chorismate product from which each pathway starts is different (Figure 2). The precursor for the second ring is another feature that differentiates these three pathways (Figure 2). Finally, specialized plant 1,4-NQs synthesized via the acetate-polymalonate pathway, as the name implies, are derived from the condensation of acetyl-CoA with multiple malonyl-CoA molecules (Figure 2).

### The OSB pathway

The OSB pathway consists of a core set of seven reactions that convert chorismate to 1,4-dihydroxy-2-naphthoate (DHNA; Figure 2), which supplies the 1,4-naphthalenoid ring for menaquinone in most bacteria and for phylloquinone in all plants. In some plants, DHNA is also the precursor for specialized 1,4-NQs, such as lawsone (2-hydroxy-1,4-NQ; Figure 2) and juglone (5-hydroxy-1,4-NQ; Figure 2). The first indication for the existence of the OSB pathway came in the 1960s when it was shown that [U-^14^C]-shikimate fed to *Escherichia coli* and to etiolated maize shoots labeled menaquinone^28^ and phylloquinone,^29^ respectively. Experiments demonstrating that labeling from [U-^14^C]-shikimate could also be retrieved in the benzene moiety (ring *A*) of lawsone^30,31^ and juglone^32^ soon followed. First evidence for the origin of the quinone moiety (ring *B*) in OSB-derived 1,4-NQs came from tracer studies in *Impatiens balsamina* (Garden balsam) showing that [2-^14^C]-glutamate^33^ and [U-^14^C]-α-ketoglutarate^34^ labeled lawsone in a specific pattern. Extension of this finding led to further investigations establishing that OSB is an intermediate and that DHNA is the product from which the OSB pathway branches toward production of various 1,4-NQs.^35–39^

Nearly all the plant OSB pathway genes have been identified and functionally characterized from biochemical and genetic studies investigating phylloquinone biosynthesis in *Arabidopsis*.^40–44^ The OSB route begins with the isomerization of chorismate to isochorismate by isochorismate synthase^41,42^ (ICS; reaction 1, Figure 2), an enzyme that is shared with plant pathways for salicylic acid^40,41,45^ and 2,3-dihydroxybenozic acid^46,47^ biosynthesis. Isochorismate is then the substrate for PHYLLO,^42^ a trifunctional enzyme with 2-succinyl-5-enolpyruvyl-6-hydroxy-3-cyclohexene-2-carboxylate (SEPHCHC) synthase, 2-succinyl-6-hydroxy-2,4-cyclohexadiene-2-carboxylate (SHCHC) synthase and OSB synthase domains (reactions 2–4, Figure 2). On the basis of biochemical characterization of their bacterial orthologs, these enzymes, respectively, are known to sequentially catalyze the addition of α-ketoglutarate^48^ (ultimately providing the succinyl side chain of OSB) to form SEPHCHC, the 2,5-elimination of the pyruvyl side chain to form SHCHC,^49^ and dehydration to produce OSB,^50,51^ the aromatized ring of which serves as the benzene moiety (ring *A*) in the eventual 1,4-naphthalenoid structure of DHNA (Figure 2). Next, the succinyl side chain of OSB is activated to its corresponding CoA-ester by OSB-CoA ligase (reaction 5, Figure 2)^43,52^ and cyclized by DHNA-CoA synthase (reaction 6, Figure 2)^53^ (formerly misnamed as DHNA synthase) to produce the quinone moiety (ring *B*) in the resulting product, DHNA-CoA (Figure 2). Although no plant DHNA-CoA synthase has been functionally characterized, a predicted ortholog of the *E. coli* DHNA-CoA synthase gene, *menB*, is present in *Arabidopsis* and co-expresses with other known phylloquinone biosynthetic genes.^43^ Phylogenetic reconstruction has revealed that plant DHNA-CoA synthases belong to the type I class, which rely on bound bicarbonate as the catalytic base,^54^ suggesting the OSB pathway may be regulated by cellular bicarbonate levels. The final step of the core OSB pathway is hydrolysis of DHNA-CoA to DHNA (Figure 2), a reaction that was previously assigned to DHNA-CoA synthase, then to SHCHC synthase and finally thought to occur spontaneously.^55,56^ Only recently was it demonstrated that cyanobacteria,^57^ *E. coli*^58^ and plants^44^ contain thioesterases catalyzing the hydrolysis of DHNA-CoA to DHNA (reaction 7, Figure 2). Once formed, DHNA is then used to synthesize phylloquinone in all plants. First, DHNA is phytylated by DHNA phytyl transferase (reaction 8, Figure 2),^59^ a reaction that is accompanied by decarboxylation and spontaneous oxidation of the 1,4-naphthalenoid ring.^60^ A demethylnaphthoquinone oxidoreductase then reduces the resulting demethylphylloquinone product to demethylphylloquinol (reaction 9, Figure 2),^61^ which is promptly transmethylated by demethylphylloquinone methyltransferase to phylloquinol (reaction 10, Figure 2),^61,62^ the reduced form of phylloquinone.

One interesting, yet poorly understood, aspect of the plant OSB pathway is its split between plastids and peroxisomes. Fluorescent protein fusion experiments revealed that the conversion of chorismate to OSB by ICS and PHYLLO occurs in plastids.^41,42^ The site(s) of OSB-CoA formation, however, remains enigmatic as fluorescent protein fusion experiments showed OSB-CoA ligase is dual localized in plastids^43^ and peroxisomes.^63^ Peptide fragments of the spinach MenB ortholog, *a priori* catalyzing the formation of DHNA-CoA from OSB-CoA, were retrieved in proteomes obtained from leaf peroxisomes, and the *Arabidopsis* ortholog was demonstrated through fluorescent protein fusion experiments to localize to peroxisomes.^63^ In *Arabidopsis*, DHNA-CoA thioesterase activity was detected in purified peroxisomes, and found to be absent in plastids.^44^ Moreover, based on fluorescent protein fusion experiments and proteomics evidence, the cognate enzymes were established to localize to peroxisomes.^44,64^ Together, these data suggest that OSB and/or OSB-CoA is exported from plastids and converted to DHNA in peroxisomes. The final three enzymes in phylloquinone biosynthesis are localized in plastids,^59,61,62^ definitively indicating that DHNA must be transported from peroxisomes to plastids. It is also likely that DHNA is needed in plastids to synthesize AQs derived from the OSB pathway (Figure 1). Labeling studies with *Rubia tinctorum*^65^ and *Cinchona* ‘Robusta’^66^ cell cultures showed that the methylerythritol 4-phosphate (MEP) pathway, which is localized in plastids, is overwhelmingly the dominant source of isopentenyl diphosphate/dimethylallyl diphosphate (DMAPP) used to synthesize ring *C* of their respective AQs (Figure 2). Similarly, labeling patterns retrieved in the anthrasesamone type AQs produced by sesame (*Sesamum indicum*) hairy root cultures fed with [1-^13^C]-glucose revealed DHNA from the OSB pathway and GPP produced by the MEP pathway as the sources of rings *A* and *B*, and ring *C*, respectively.^67^ These studies are in agreement with those performed by Leistner showing that [2-^14^C]- and [5-^14^C]-mevalonic acid are negligibly incorporated into ring *C* of alizarin (Figure 1b), a red pigment produced in roots of madder (*R. tinctorum*).^68,69^ It still remains an open question if there are additional subcellular destinations for DHNA in plants as, as described below, none of the specialized 1,4-NQ biosynthetic enzymes downstream of DHNA have been identified.

In many members of the Juglandaceae, including black walnut (*Juglans nigra*) and English walnut (*Juglans regia*), DHNA is an intermediate in the synthesis of juglone and several other related 1,4-NQs.^70^ Chemical degradation of juglone isolated from *J. regia* leaves fed with radiolabeled precursor revealed that the carboxyl group of shikimate is equally distributed between the keto groups (C1 and C4) in the quinone moiety of juglone, leading to the hypothesis that a symmetrical intermediate like 1,4-naphthoquinone (Figure 2) must be an intermediate in the pathway.^32^ Indeed, 1,4-naphthoquinone was found to be present in *J. regia* leaves and to be labeled by radiolabeled OSB.^39,71^ This suggests the existence of an enzyme that decarboxylates DHNA to 1,4-naphthoquinone. The subsequent conversion of 1,4-naphthoquinone to juglone is likely to be carried out by a hydroxylase, perhaps belonging to the cytochrome P450^72^ or 2-oxoglutarate/Fe(II)-dependent dioxygenase (2-ODD)^73^ families.

Phenolic compounds are often glycosylated to increase their solubility and stability, to aide in transport and sequestration, and to render the compounds physiologically inactive in plants.^74^ It should come as no surprise then that in several species, such as black and English walnut,^75^ *J. major*, *J. microcarpa*^76^ and a number of pecan (*Carya illinoensis*) cultivars,^77,78^ juglone has been found to accumulate in many tissues in its glycosylated form, hydrojuglone glucoside (HJG; 1,5-dihydroxy-4-naphthalenyl-β-D-glucopyranoside; Figure 1b). This modification may allow juglone to be stored in large quantities and to reduce the potential for autotoxicity. In English walnut leaves, glycosyltransferase activity with a benzoquinone substrate has been detected,^76^ though the responsible enzyme is unknown and activity with HJG has yet to be demonstrated. Moreover, glycosylation depends on the quinone substrate being in its reduced form,^76^ thus implicating the existence of an oxidoreductase capable of first reducing juglone to hydrojuglone (1,4,5-trihydroxynaphthalene; Figure 1b). Although there have been no reports on the presence of such an enzyme in *Juglans* species, a quinone oxidoreductase that uses NADH or NADPH as electron donors to reduce a variety of quinones, including juglone, has been identified in roots of the parasitic plant *Triphysaria versicolor*.^79^ Just as reduction followed by glycosylation inactivates juglone, deglycosylation of HJG by an unknown β-glucosidase purified from English walnut husks releases hydrojuglone aglycone, which then spontaneously oxidizes to generate active juglone.^80^

Another major specialized 1,4-NQ derived from DHNA is lawsone, the molecule responsible for the reddish-orange dyestuff extracted from Henna (*Lawsonia inermis*) leaves. Lawsone is also found in the flowering aerial parts and roots of several *Impatiens* species.^81–83^ Similar to juglone, lawsone is a simple hydroxylated 1,4-NQ, though its biosynthesis is quite different. This idea first emerged after feeding studies with *I. balsamina* showed that labeled shikimate incorporated into juglone and lawsone in different patterns.^31,32^ Later, using the same model species, Chung *et al.*^71^ demonstrated with stable-isotopically labeled [1-^13^C]-OSB that the C1 keto group of lawsone was substantially more highly labeled compared with the C4 keto group, indicating that OSB is asymmetrically incorporated into lawsone.^71^ Therefore, in contrast to juglone, the biosynthesis of lawsone does not proceed through a symmetrical 1,4-naphthoquinone intermediate and is instead likely formed via oxidative decarboxylation of DHNA by an unknown enzyme (Figure 2). The glucosylated form of reduced lawsone, 1,2,4-trihydroxynaphthalene-1-*O*-glucoside (Figure 1b), has been reported in *Impatiens glandulifera* (Himalayan balsam), thus pointing to the presence of an oxidoreductase and a glycosyltransferase analogous to those involved in metabolizing juglone.

Lawsone is also precursor to other 1,4-NQ natural products. An allelopathic methylated lawsone derivative, 2-methoxy-1,4-NQ (2-MNQ; Figure 2), is found in many tissues of several *Impatiens* species,^81,83–85^ and is almost certainly formed via an *S*-adenosylmethionine-dependent *O*-methyltransferase.^86^ In several native Central and South American trees, including the Pau d’arco tree (Red Lapacho; *Tabebuia impetiginosa*, syn. *Tabebuia avellanedae*)^87^ and *Tabebuia guayacan*,^15^ lawsone is proposed to provide the hydroxylated naphthalenoid structure of the prenylated 1,4-NQ lapachol (Figure 2).^16^ The identity of the responsible prenyltransferase is unknown and it is still unclear if the DMAPP moiety of lapachol is predominantly derived from the MEP or MVA pathway. Lapachol is also thought to be a precursor for other 1,4-NQ, 1,2-NQ (for example, β-lapachone) and AQ derivatives (Figure 2) contributing to the resistance of *Tabebuia* trees to marine borers^88,89^ and to their wide range of medicinal properties.^16,87^

### The 4HBA/MVA pathway

Many boraginaceous species utilize the 4HBA/MVA pathway to synthesize a subclass of 1,4-NQs called isohexenylnaphthazarins (IHNs). These compounds are comprised of a NZ ring (Figure 1b) conjugated with a lipophilic side chain on the quinone moiety. The IHNs encompass the red-pigmented compounds shikonin, alkannin and at least 40 other acylated derivatives (Figure 2) synthesized in roots of medicinal species like *Lithospermum erythrorhizon* and *Alkanna tinctoria*.^90^ Early tracer experiments demonstrated that phenylalanine (via cinnamic acid and 4HBA) and mevalonic acid are precursors for the benzene and quinone rings, respectively, of alkannin produced in *Plagiobothrys arizonicus*.^91^ This finding, in combination with isolation of 3-geranyl-4HBA and geranylhydroquinone from cell cultures of *L. erythrorhizon*,^92,93^ led to the hypothesis that alkannin and shikonin are likely synthesized via a pathway analogous to ubiquinone biosynthesis with subsequent ring closure reactions (Figure 2).

Biosynthesis of benzoic acids from phenylalanine in plants involves a complex network of metabolic routes branching off the core phenylpropanoid pathway.^24^ Administration of the phenylalanine-ammonia lyase (PAL) inhibitor aminoindan-2-phosphonic acid to *L. erythrorhizon* cell cultures effectively blocked shikonin formation.^94^ Yazaki *et al.*^95^ demonstrated that the enzymatic formation of 4HBA from 4-coumaric acid (4CA) in *L. erythrorhizon* cell cultures partially proceeds through the ‘non-oxidative route’ based on the presence of a 4-hydroxybenzaldehyde intermediate, dependence on NAD and the lack of an ATP or CoA requirement.^95^ It was later discovered that *L. erythrorhizon* cell cultures are also capable of converting 4CA-CoA to 4HBA via the ‘β-oxidative route’,^96^ which was recently shown to provide the 4HBA ring for ubiquinone biosynthesis in *Arabidopsis*.^97^ Genes encoding the core phenylpropanoid pathway enzymes PAL, cinnamic acid 4-hydroxylase (C4H) and 4CA-CoA ligase (4CL) have been cloned and studied from *L. erythrorhizon*^98,99^ and *Arnebia euchroma*,^100^ but those involved in the ‘non-oxidative’ and ‘β-oxidative’ routes have not. Benzoic acid ‘β-oxidative route’ genes have been identified in other species, while all but one of the ‘non-oxidative route’ genes remain unknown across all plants.^24^ Once synthesized, 4HBA can be glucosylated by a cytosolic glucosyltransferase and stored in the vacuole until released to its free form by a cytosolic β-glucosidase upon stimulation of shikonin production.^101^

In plants, GPP is predominantly synthesized from isopentenyl diphosphate and DMAPP derived from the MEP pathway using a GPP synthase (GPPS) localized in plastids.^25^ It is therefore noteworthy that the GPP precursor ultimately providing ring *B* of shikonin- and alkannin-type 1,4-NQs was shown to be derived from the MVA pathway (based on labeling^91^ and inhibitor studies^94,100^) and to originate via the only known cytosolically localized GPPS (reaction 11, Figure 2).^102,103^ Expression of multiple MVA pathway genes in *A. euchroma*,^100^ and the activity and cognate gene expression of the key MVA pathway enzyme 3-hydroxy-3-methylglutaryl-CoA reductase (HMGR) in *L. erythrorhizon*,^104^ correlate with shikonin production. Generally, MVA pathway genes are more highly expressed in non-photosynthetic tissues, like roots, whereas MEP pathway genes are primarily active in green tissues.^25^

The committed step of shikonin and alkannin biosynthesis begins with the addition of GPP to 4HBA by a GPP:4HBA 3-geranyltransferase (*p*-hydroxybenzoate:geranyltransferase, PGT; reaction 12, Figure 2). Activity of this enzyme was first reported in *L. erythrorhizon* extracts.^105^ Later, PGT was shown to be localized to the endoplasmic reticulum^106^ and to have a high affinity for GPP (*K*_m_=18.4 μM) and 4HBA (*K*_m_=13.8 μM).^107^ Two cDNAs encoding PGTs with 93% identity were isolated from *L. erythrorhizon* cell cultures.^108^ Subsequent biochemical characterization of one isoform, LePGT1, revealed that the N-terminal 130 amino acids are responsible for its specificity for GPP^109^ and that it is inhibited by aromatic substrates with two phenolic hydroxyl groups.^110^

After the PGT-catalyzed reaction, very little is known about the biosynthesis of shikonin- and alkannin-type 1,4-NQs. It is likely the next steps entail decarboxylation and hydroxylation of the C1 position of the 3-geranyl-4HBA product of PGT. In bacterial ubiquinone biosynthesis, the C1 position of the polyprenylated 4HBA product is non-oxidatively decarboxylated using a prenylated flavin cofactor to produce a prenylphenol intermediate,^111,112^ which is hydroxylated further down the pathway.^113^ It is possible that 3-gernanyl-4HBA is decarboxylated and hydroxylated in a similar fashion by discrete enzymes to produce geranylhydroquinone (GHQ), which has been detected *in planta*,^92,93^ via a 2-geranyl-phenol intermediate (Figure 2). Alternatively, 3-geranyl-4HBA may be directly converted to GHQ by oxidative decarboxylation. The next reaction in the pathway is hydroxylation of the GHQ isoprenoid side chain to produce 3″-hydroxy-GHQ by a GHQ 3″-hydroxylase (reaction 13, Figure 2). A GHQ 3″-hydroxylase was partially purified from the microsomal fraction of *L. erythrorhizon* cell cultures and shown to require NADPH and molecular oxygen as cofactors, suggesting it is a cytochrome P450-dependent monooxygenase.^114^ Moreover, the purified GHQ 3″-hydroxylase was found to have a *K*_m_ for GHQ of 1.5 μM and to be inhibited by the shikonin derivative acetylshikonin at a concentration as low as 10 μM.^114^ It has been proposed that cyclization of 3″-hydroxy-GHQ to form the quinone moiety of the 1,4-NQ skeleton occurs via oxidation of the C3″ position to generate an aldehyde capable of forming the aromatic nucleus via an electrophilic reaction.^114^ As deoxyshikonin has been detected in shikonin-producing species,^115,116^ it is probable that it is the final intermediate in the pathway and is converted to shikonin (or alkannin in alkannin-producing species) by GHQ 3″-hydroxylase or a similar enzyme. To date no enzymes downstream of GHQ have been identified.

Production of shikonin and its derivatives is influenced by many external factors, as has been previously summarized,^117–120^ and is in large part modulated by transcriptional regulation of metabolic genes.^100,121,122^ Biosynthesis was also shown to be controlled by auxin,^123^ methyl jasmonate^124^ and ethylene.^125–127^ Overexpression of the *L. erythrorhizon MYB1* (*LeMYB1*) transcription factor gene, an ortholog of the *Nicotiana tabacum MYB* involved in regulating phenylpropanoid metabolism,^128^ led to increased expression of *PAL*, *HMGR* and *PGT*.^129^ Non-biosynthetic regulators of shikonin production have also been identified in *L. erythrorhizon*, including an unknown cell wall protein, LePS-2, perhaps involved in deploying shikonin,^130^ and LeDI-2, a small hydrophobic dark-inducible protein of unknown function.^131^ Downregulation of *LeDI-2* reduced the shikonin pool size, though had no affect on the expression of *PAL* or activity of PGT.^131^ In addition to increasing expression of biosynthetic genes, *LeMYB1* overexpression also increased expression of the shikonin regulators *LeDI-2* and *LePS-2*.^129^

### The HGA/MVA pathway

The HGA/MVA pathway (also referred to as the toluhydroquinone or toluquinol pathway) is widely distributed throughout, although limited to, plants within the Pyroloideae subfamily of the Ericaceae. Very little progress has been made toward understanding the metabolism of this pathway as labeling experiments in *Chimaphila umbellata* (pipsissewa) established that tyrosine^132^ and DMAPP derived from the MVA pathway^133^ provide precursors for chimaphilin (2,7-dimethyl-1,4-NQ; Figure 2). One unique feature of the HGA/MVA pathway is that shikimate (via tyrosine) ultimately provides the quinone moiety of the 1,4-naphthalenoid ring, compared with the OSB and 4HBA/MVA pathways in which shikimate (via isochorismate and phenylalanine, respectively) provides the benzene moiety (Figure 2).

Bolkart and Zenk showed that the β-carbon atom of tyrosine is exclusively incorporated into the 2-methyl position of chimaphilin via homogentisate and toluquinol intermediates,^132,134^ and that the C7 methyl group arises from the C2 of mevalonic acid.^133^ It can thus be envisioned that upon decarboxylation of homogentisate, which is also precursor for tocochromanols and plastoquinone synthesized in plastids (recently reviewed^135^), DMAPP is attached to the toluquinol product (Figure 2). The resulting prenylated intermediate, dimethylallyl-toluquinol, would then be cyclized to 5,8-dihydro-2,7-dimethylnaphthalene-1,4-diol, which would subsequently be aromatized to produce chimaphilin (Figure 2). Support for such a biosynthetic pathway architecture comes from the isolation of toluquinol, its glucoside (homoarbutin) and the glucoside of 5,8-dihydro-2,7-dimethylnaphthalene-1,4-diol (renifolin) from *Pyrola media* and *Pyrola incarnata.*^136–138^ Beyond supporting the postulated pathway leading to chimaphilin, these findings raise questions about the role of glycosylation in the HGA/MVA pathway. Similarly, *Moneses uniflora* produces chimaphilin derivatives, including 8-chlorochimaphilin, 8-hydroxychimaphilin and 3-hydroxychimaphilin, that also appear to be subject to glycoslylation in their reduced forms.^139^

### The acetate-polymalonate pathway

A fourth route to synthesize plant 1,4-NQs, the acetate-polymalonate pathway (also referred to as the polyketide pathway), relies on CoA-linked acetate and malonate substrates, occurs throughout at least half a dozen unrelated families,^7^ and is most notably responsible for the production of plumbagin (5-hydroxy-2-methyl-1,4-NQ), droserone (3,5-dihydroxy-2-methyl-1,4-NQ), 5-*O*-methyldroserone and 7-methyljuglone (Figure 2), as well as bis-1,4-NQs, such as chitranone and diospyrin (Figure 1b).^140^ Support for the acetate-polymalonate pathway surfaced from experiments conducted by Durand and Zenk showing that labeled acetate precursors were incorporated into plumbagin in young *Plumbago europaea* shoots^141^ and *Drosophyllum lusitanicum* leaves.^142^ Moreover, it was revealed that neither shikimate- nor methionine-labeled plumbagin strongly indicating that its synthesis (and by extension, the synthesis of droserone, 5-*O*-methyldroserone and 7-methyljuglone) does not proceed through a juglone intermediate derived from the OSB pathway (Figure 2).^142^

On the basis of stable-isotope feeding experiments in *Triphyophyllum peltatum* callus cultures, which confirmed the acetogenic origin of plumbagin and droserone, Bringmann *et al.* proposed the involvement of polyketide synthases (PKSs) in the acetate-polymalonate pathway.^143,144^ In plants, PKSs belong to the type III class, which catalyze C–C bond formation in a single active site through a series of decarboxylation, condensation and cyclization reactions using a CoA-ester substrate (for example, acetyl-CoA) and CoA-ester extenders (for example, malonyl-CoA).^26^ Recently, cDNAs encoding type III PKSs (reaction 14, Figure 2) from *Plumbago indica* roots and *D. lusitanicum* calluses have been isolated.^145,146^ Biochemical characterization of the cognate recombinant enzymes revealed that both were capable of accepting acetyl-CoA (*K*_m_=31 μM for *D. lusitanicum* PKS) as starter and catalyzing multiple sequential decarboxylative condensations with malonyl-CoA (*K*_m_=83 μM for *D. lusitanicum* PKS).^145,146^ However, under the tested *in vitro* conditions, neither formed expected naphthalene products and instead produced α-pyrones, which may have been an artifact given the absence of pyrone metabolites in the tissues from which the cDNAs were isolated.^145,146^ Together with the lack of other PKS candidates and the high plumbagin content in the tissues examined, it is likely the identified PKSs provide the 1,4-NQ backbone via the postulated intermediates depicted in Figure 2.^145,146^ The acetate-polymalonate pathway is also likely to rely on a polyketide reductase to remove the oxygen atom of the third acetate unit before the initial cyclization.^146^

That plumbagin, droserone and 5-*O*-methyl droserone have been described to co-occur in *Nepenthes* species^147,148^ suggests these compounds may be part of a linear biosynthetic pathway as depicted in Figure 2. The postulated naphthalene intermediate is likely oxidized to generate either 7-methyljuglone or plumbagin, an idea consistent with the fact that many Droseraceae species exclusively contain one or the other of these 1,4-NQs.^149,150^ Perhaps using enzymes belonging to the same classes describuned above for juglone, lawsone and 2-MNQ synthesis, plumbagin may be further oxidized to produce droserone, and then methylated at the C5 hydroxyl group to generate 5-*O*-methyldroserone (Figure 2). Similar to other aforementioned 1,4-NQs, acetate-polymalonate-derived 1,4-NQs are subject to modifications, including glycosylation.^151,152^ Certain types of AQs, such as emodin produced by rhubarb and related species,^153,154^ are also synthesized via PKSs with acetyl-CoA and malonyl-CoA precursors. However, their biosynthesis does not proceed through a 1,4-NQ like those derived from the OSB pathway, so they will not be covered in this review. Generally, AQs synthesized via PKSs contain modifications in both rings *A* and *C*, while those derived from the OSB pathway (for example, alizarin) only contain functional groups on ring *C*, although exceptions exist.^66^

## Biochemical perspectives on the functions of specialized 1,4-NQs produced by horticultural species

**Figure 3:**
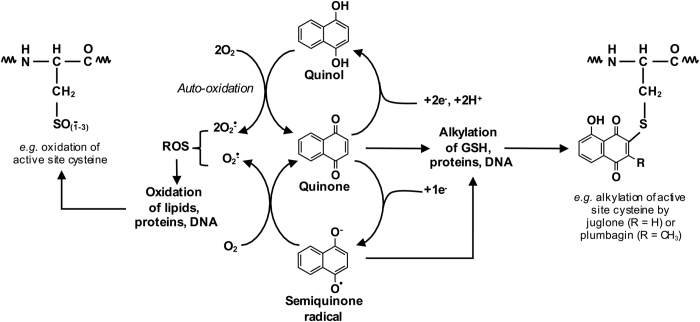
Potential mechanisms of action for 1,4-NQs. Plant 1,4-NQ redox cycling may lead to the generation of reactive oxygen species (ROS), which can oxidize certain cellular macromolecules. The quinone and/or semiquinone forms of plant 1,4-NQs can react with nucleophiles to form adducts.

An understanding of the ecological significance of 1,4-NQ production in horticultural plants requires recognition of the biochemical properties of quinones in general and 1,4-NQs in particular. Quinones undergo redox cycling and alkylation reactions, generating reactive oxygen species (ROS) and adduct formation with proteins and DNA.^155^ Alkylation (also termed arylation) of reduced glutathione (GSH) or cysteine residues of proteins is particularly common, leading to depletion of GSH levels and/or protein cysteine chemical modifications.^155,156^ The one- or two-electon reduction of quinones to the semiquinone radical or quinol, respectively, leads to their autoxidation by molecular oxygen to the superoxide anion radical (O_2_^−^), which subsequently disproportionates into O_2_ and H_2_O_2_, promoting oxidation of lipids, proteins and DNA (Figure 3).^155^

In the case of 1,4-NQs, the cellular reduction of the quinone moieties in mammalian cells can be mediated by cytochrome P450 reductase, forming the semiquinone, or by NAD(P)H: quinone oxidoreductase-1 (NQO-1, DT-diaphorase), forming the quinol. Thus, both reactions may occur at the expense of NADH or NADPH. Quinols may undergo detoxification via coupling of the hydroxyl moieties to water-soluble molecules,^157^ such as glycosylation as described above for juglone,^80^ lawsone,^83^ 7-methyljuglone and droserone.^152^ It is tempting to speculate that such quinol conjugates are the predominant forms *in planta* and afford protection against autotoxicity. However, naphthoquinone glycosides in the genus *Drosera* usually appear only as minor components of the total naphthoquinone pool.^18^ Moreover, the 1,4-NQ glucosides rossoliside and plumbaside A, isolated from *Nepenthes*, showed no incorporation after feeding of either [U-^13^C_2_]-sodium acetate or [U-^13^C_3_,^15^N]-alanine, suggesting these glycosides are storage forms with very low turnover rates.^158^

Depending on ring modifications, some 1,4-NQs are also good electrophiles that react with nucleophiles, including cysteine residue thiol groups in some proteins, to form adducts in cells (Figure 3).^156,157^ Such alkylation reactions can occur when a free C3 position is present in the 1,4-naphthalenoid structure, though the C2 substituent must also allow access and sufficient electrophilicity.^157^ For example, lawsone, which has a free C3 position, but a hydroxyl group at C2 (Figure 2), is considered a weak alkylating agent compared with juglone (free C2 and C3 positions; Figure 3) and plumbagin (free C3 position and methyl group at C2; Figure 3).^157^ The type and placement of functional groups in the benzene moiety also influences the bioreactivity and cytotoxicity of 1,4-NQs. Juglone, plumbagin, NZs and other 1,4-NQs with at least one hydroxyl group in the benzene moiety are more potent topoisomerase inhibitors compared with unsubstituted 1,4-NQs and 1,4-NQs hydroxylated on the quinone moiety (for example, lawsone).^159,160^

Given the aforementioned reactivity of 1,4-NQs in biological systems, it should not be surprising that 1,4-NQs have frequently been observed to induce marked perturbations of metabolism, including ROS production, thiol depletion, alkylation/arylation of numerous target proteins, DNA damage and genotoxicity in diverse biological systems. Specific examples of these effects, induced by 1,4-NQ natural products derived from horticultural species, in plants, microorganisms, insects and mammals are described below, with particular emphasis on potential alkylation/arylation target sites.

### Plant–plant interactions (allelopathy)

Allelopathy is the term used to describe the harmful effect one plant exerts on another via the release of natural products into the environment.^161^ This is the definition that will be adopted in the remainder of this review, though it is recognized that allelopathy is also used to generally describe any direct or indirect effect, beneficial or harmful, one plant has another plant.^162^

The classic example of allelopathy is the release of the phytotoxic 1,4-NQ, juglone, from black walnut.^163,164^ Other 1,4-NQ-producing plants reported to exhibit allelopathy and notable as noxious invaders include *Echium plantaginerum* (Paterson’s curse; produces shikonin and its derivatives)^165^ and *I. glandulifera* (producer of lawsone and 2-MNQ).^85,166^ Undoubtedly, secretion of 1,4-NQs from the roots and/or leaching of 1,4-NQs from leaves and leaf litter may have contributed to the ecological success of these invading species via their phytotoxic effects on native species by the general mechanisms described above (that is, ROS production, GSH depletion and/or alkylation of proteins and DNA of neighboring plants). The best studied plant-derived phytotoxic 1,4-NQs are plumbagin and juglone, which induce ROS production in tobacco BY-2 cells, ultimately resulting in programmed cell death.^167^ In lettuce, juglone induces oxidative damage to the root apical meristem via ROS and a cascade of cellular changes, including decreased mitochondrial potential, chromatin condensation and DNA fragmentation.^168^ Juglone also triggers a large number of changes in gene transcription associated with cell growth, cell wall formation, chemical detoxification and abiotic stress responses via rapid induction of ROS in rice roots.^169^

Juglone’s specific cellular alkylation/arylation protein targets have been less intensively investigated in plants than in mammalian systems (see Pharmacology section below), but a number of potential targets can be postulated from the literature. Rapid irreversible growth inhibition of maize coleoptiles and inhibition of auxin-induced growth in maize coleoptile segments strongly suggests that the plasma membrane H^+^-ATPase may be a juglone alkylation/arylation target.^170^ Direct arylation of cysteine residues of jack bean urease by juglone (but not by lawsone) has been reported.^171^ Juglone is also a potent inhibitor of *Malus domestica* MdPin1, a homolog of a phosphorylation-specific peptidyl prolyl *cis*/*trans* isomerase (PPIase) in humans called Pin1 that has an important role in cell cycle regulation.^172^

The molecular mechanisms governing the large interspecies variability within the plant kingdom with respect to the toxic effects of juglone summarized by Willis^164^ are virtually unexplored. It is still unclear whether species unaffected by juglone are equipped with enzymes that facilitate detoxification and/or proteins that regulate juglone exclusion, transport and/or sequestration/compartmentation.

### Plant–microbe interactions

The toxicity of plant-derived 1,4-NQs toward various bacteria, fungi and other microorganisms is widely recognized. Examples of the growth inhibitory effects that plant 1,4-NQs have on microorganisms documented to be associated with oxidative stress and/or disrupted thiol metabolism in the target organisms include lawsone on *E. coli*;^173^ juglone on *Staphylococcus aureus*^174^ and *Acanthamoeba castellanii*;^175^ plumbagin on *Candida albicans* and *S. aureus*;^176^ 7-methyljuglone on *Mycobacterium tuberculosis*;^177^ and shikonin on *Candida albicans*.^178^

Reddy *et al.*^179^ investigated the antimicrobial effects of plumbagin in *Bacillus subtilis* by identifying differentially expressed proteins, and found evidence suggesting the 1,4-NQ represses the tricarboxylic acid cycle, the electron transport chain and the fatty acid synthesis; however, specific arylation targets have yet to be defined in this organism. Juglone inactivates the *E. coli* PPIase by covalent modification of cysteine residues.^180^ In yeast, juglone may not only inhibit the PPIase homolog, ESS1 but also RNA polymerase II, most likely by modification of sulfhydryl groups.^181^

The toxicity of plant 1,4-NQs to human pathogens (for example *M. tuberculosis*) is of particular interest and has obvious overlaps with the Pharmacology section below. In *M. tuberculosis*, 7-methyljuglone is a subversive substrate for mycothiol disulfide reductase.^177^ Moreover, 7-methyljuglone and its bis form, diospyrin (Figure 1b), produced by *Diospyros montana*,^182^ are potent inhibitors of DNA gyrases of *M. tuberculosis*, *E. coli* and *S. aureus*.^183^ DNA gyrase is a DNA topoisomerase that is present in bacteria and plants, but not in animals, and has been widely exploited as a target for antimicrobial chemotherapy.^183^ Whereas animal DNA topoisomerase of the type II (topo II) class are thought to be inhibited by formation of cysteine adducts with quinones in the N-terminal (ATPase) domain, the inhibition of DNA gyrase may be different. The naphthoquinone-binding site is within the N-terminal domain of GyrB, but as one of the enzymes examined (*S. aureus* gyrase) lacks Cys residues in the ATPase domain, it is unlikely that covalent adducts with Cys residues form part of the mode of binding.^183^ Moreover, there was no evidence of adduct formation with *M. tuberculosis* gyrase.^183^ Microarray analysis of *M. tuberculosis* in response to plumbagin challenge identified 103 and 171 up- and downregulated genes, respectively, but it is presently unknown whether these transcriptional responses were the sole consequence of DNA gyrase inhibition.^184^

Being that plants are susceptible to pathogens, it is tempting to speculate that the production of 1,4-NQs by plants may not only serve a role in allelopathy (above) but also in plant disease defense. Supporting this notion, juglone has been shown to be a potent and specific inhibitor of the growth of the fire blight pathogen, *Erwinia amylovara*.^185^ Similarly, it was reported that plumbagin is a potent growth inhibitor of a number of phytopathogenic fungi.^186^

Certain soil bacteria, such as *Pseudomonas putida*,^187^ are capable of degrading juglone, although little is understood about the role juglone has in shaping the soil microflora. It has been proposed that arbuscular mycorrhizal fungal hyphae may have a key role in transporting juglone in the rhizosphere.^188,189^ Hook *et al.* argue that because the production of antimicrobial 1,4-NQs is often restricted to specific root cells and elicited by soil-borne microbes, this suggests their role is in plant defense at the cellular level in the rhizosphere.^20^

The production of 1,4-NQs by many carnivorous plants (especially in the family Droseraceae)^150^ may have a key role in maintaining sterility of the digestive fluids secreted by these organisms, an idea that has been speculated for *Nepenthes* sp. (pitcher plants).^148,190^ Plumbagin secreted by the Venus fly trap (*Dionaea muscipula*) also has antimicrobial activity against food-related pathogenic and putrefactive bacteria.^191^ Perhaps more significantly, the chemical composition of the *Nepenthes* pitcher fluid may promote a specific microbiome dedicated to chitinolytic, proteolytic, amylolytic, and cellulolytic and xylanolytic activities for digesting pitcher-captured insects.^192^ Resistance mechanisms used by pitcher-associated microbes to pitcher plant 1,4-NQs remain unexplored. Clues to the molecular mechanism of bacterial plumbagin resistance have come from studies with *E. coli* demonstrating that two plumbagin-responsive genes *ygfZ* and *sodA* are required for counteracting toxicity.^193^ Furthermore, it was found that Cys228 in YgfZ is needed for the degradation of plumbagin, which may be excreted in a methylated and less-toxic form.^193^

### Plant–insect interactions

The toxicity of plant-derived 1,4-NQs toward insects has also been documented. It appears that plumbagin, juglone and 2-MNQ may specifically target ecdysone 20-monooxygenase, an enzyme responsible for converting the molting hormone ecdysone to its more physiologically active metabolite, 20-hydroxyecdysone.^194,195^ Plumbagin is also an inhibitor of chitin synthetase^194^ and, similar to other 1,4-NQs, is an effective anti-feedant defensive agents against insects.^195–197^ It is also plausible that certain insects that have gained the ability to feed on 1,4-NQ-producing plants may utilize these molecules for their own defense; however, the precise mechanisms of tolerance of these organisms has been underexplored.^198^ Piskorski *et al.*^199,200^ propose that the larvae of the codling moth, *Cydia pomonella*, are able to survive on walnut trees by excreting hydrojuglone in their frass.^199,200^ However, this hypothesis is difficult to reconcile with the observation that hydrojuglone rapidly autoxidizes to juglone in air.^80^

Insectivorous plants face a dilemma in both attracting insects for pollination and as prey. The sundew, *Drosera auriculata*, maintains a distinct profile of volatiles emitted from flowers in comparison with their sticky traps, with plumbagin restricted to the trap, suggesting a possible role for plumbagin in attracting insect prey with volatile preferences that are distinct from insect pollinators.^201^ It has been suggested that plumbagin may contribute to oxidative protein modification as a predigestive mechanism in Venus fly trap.^202^ Plumbagin production has also been observed to be induced in response to chitin in *Drosophyllum lusitanicum* suspension cultures.^203^ In *Nepenthes khasiana*, chitin specifically induces production of droserone and 5-*O*-methyl droserone to protect pitcher fluid against microbes brought by visiting prey and perhaps to act as molecular triggers in prey capture and digestion.^147^

### Pharmacology (plant–human interactions)

Humans have exploited plants producing 1,4-NQs for centuries as wound-healing agents. In part this may be a function of the antibacterial and antifungal properties of 1,4-NQs discussed above, preventing opportunistic wound infections. Thomson^204^ and Hook *et al.*^20^ have reviewed the numerous bioactive properties of plant-derived NQs and discuss their cytotoxic, anticancer, antibacterial, antifungal, anti-inflammatory and anti-parasitic activities. These and other reviews on the pharmacological properties of individual 1,4-NQ-producing species, including Henna,^205–208^ *Plumbago seylanica*^209^ and Venus fly trap,^210^ or individual 1,4-NQs of plant origin, such as the alkannins and shikonins,^90,211–214^ plumbagin and its analogs,^215^ 7-methyljuglone,^216^ and laphachol,^16^ demonstrate the considerable pharmacological interest in this class of molecules in recent years.

As described above for other biological systems, plant-derived 1,4-NQs can elicit oxidative stress and disrupt thiol metabolism in mammalian cell systems, including juglone in melanoma (B16F1) tumor cells;^217^ juglone, shikonin and plumbagin in rat liver microsomes;^218^ juglone and plumbagin in HaCaT human keratinocytes;^219,220^ plumbagin in human prostate cancer cells;^221^ and shikonin in human glioma cells,^222^ thyroid cancer cells^223^ and leukemia HL-60 cells.^224,225^ Beyond these effects, it is also evident that plant 1,4-NQs have specific effects on downregulating central mediators of mammalian inflammation.^156^ Plant-derived 1,4-NQs inhibit Kelch-like enoyl-CoA-hydratase (ECH) associated protein 1 (Keap1) most likely by alkylation/arylation of key cysteine residues in the protein.^156,157^ In addition, it has been proposed that 1,4-NQs promote glutathionylation of Keap1.^226^ This may be due in part to accumulation of oxidized glutathione (GSSG),^227^ by GSH-mediated *S*-transarylation,^228^ and/or via upregulation of glutathione *S*-transferase pi, which potentiates *S*-glutathionylation of Keap1.^229^ Regardless of the precise mechanism of Keap1 cysteine modifications, it is well established that these modifications collectively alter the binding of Keap1 to transcription factor Nrf2 (NF-E2-related factor-2). Normally, Keap1 sequesters Nrf2 in the cytoplasm and bridges it to a ubiquitin ligase, cullin 3 (Cul3), to facilitate proteasomal Nrf2 degradation.^157^ Disruption of the Keap1–Nrf2 interaction by 1,4-NQs results in Nrf2 accumulation, leading to Nrf2 nuclear transport and induction of genes containing ‘antioxidant response elements’ (AREs).^157^ Consistent with this, plumbagin and shikonin strongly activate Nrf2–ARE signaling, and were found to activate genes encoding heat shock proteins.^230,231^ The Keap1–Nrf2–ARE pathway has now emerged as a promising target to develop drugs that upregulate expression of ARE-controlled cytoprotective oxidative stress response enzymes to treat a number of diseases and conditions.^232^

Nrf2 not only regulates oxidative/xenobiotic stress response but also represses inflammation by opposing transcriptional upregulation of a number of pro-inflammatory cytokine genes.^233^ Specific anti-inflammatory effects of plumbagin appear consistent with inhibiting the activation of the transcription factor nuclear factor-κB (NF-κB)^234^ in lymphocytes,^235^ macrophages and liver cells.^236^ The underlying mechanism of plumbagin and shikonin inhibition of NF-κB activation appears to entail suppression of an inhibitor of κBα (IκBα) phosphorylation and degradation, thus precluding the phosphorylation of the p65 subunit of NF-κB.^237–241^ However, it is still unclear whether the inhibition of NF-κB activation by plumbagin, shikonin and 1,4-naphthoquinone (see ^ref. 226^) is solely mediated via Keap1-dependent activation of Nrf2/ARE signaling or by other mechanisms, especially considering Keap1-independent mechanisms of regulating Nrf2 have been well documented^227^ Furthermore, it is well established that 1,4-NQs are inhibitors of topoisomerases.^242–249^ Topoisomerase 1 (Top 1) inhibition is known to suppresses inflammatory genes, including tumor necrosis factor-α (TNF-α), and to protect against lethal inflammation *in vivo*.^250^ Thus, it seems plausible that anti-inflammatory effects of 1,4-NQs may also be mediated by Top 1 inhibition.

Similar to the *M. domestica*, yeast and *E. coli* PPIases described above, human Pin1 is inhibited by juglone.^180,251^ Pin1 is now recognized as a molecular switch for TNF-α-induced priming of the NADPH oxidase in human neutrophils,^252^ as a modulator of the type 1 immune response of T cells^253^ and as an enhancer of the oncogenic activity of the Rel proteins in the NF-κB family.^254^ Therefore, inhibition of Pin1 by juglone may contribute to the anti-inflammatory and anticancer actions of this molecule. Additional pharmacological mechanisms of action for plant-derived 1,4-NQs are listed in Table 2.

The bewildering array of actions elicited by plant 1,4-NQs listed in Table 2 are likely just the tip of the iceberg. Proteomics studies by Lame *et al.*^284^ with ^14^C-labeled 1,4-naphthoquinone indicate that this molecule targets a number of different proteins in human bronchial epithelial cells, including nucleophosmin, galectin-1, protein disulfide isomerase (PDI) and probable PDI, 60 kDa heat shock protein, mitochondrial stress-70 protein, epithelial cell marker protein and S100-type calcium-binding protein A14. A quantitative proteomic study using stable-isotope labeling by amino acids in cell culture revealed that there were at least 1225 and 267 proteins interacting with plumbagin and 341 and 107 signaling pathways and cellular functions potentially regulated by plumbagin in human PC-3 and DU145 prostate cancer cells, respectively.^285^ These proteins and pathways have critical roles in the regulation of cell cycle, apoptosis, autophagy, epithelial to mesenchymal transition and ROS generation.^285^

The diversity of targets and mechanisms of action of plant 1,4-NQs have stimulated great pharmacological interest, particularly in the area of ROS initiation and signaling, cancer therapeutic strategies and as anti-inflammatory agents.^214,286^ Moreover, numerous analogs of 1,4-NQs have been designed and synthesized to enhance their toxicity toward specific human cancer cell lines,^277,287–289^ specific proteins (for example, Hsp90),^290^ selected pathogenic organisms (for example, *Trypanosoma* sp)^291–293^ and insects.^294^

## Conclusions and future prospectives

Plant 1,4-NQs are a diverse class of metabolites possessing a wide range of ecological functions contributing to plant fitness, particularly in the horticultural species highlighted in this review (Table 1). At least four different metabolic pathways to synthesize 1,4-NQs exist in the plant kingdom. The OSB route is present in all plants to produce phylloquinone, though some species are capable of synthesizing additional 1,4-naphthalenoid natural products branching off this pathway. The 4HBA/MVA, HGA/MVA and acetate-polymalonate pathways are each restricted to certain families. Regardless of the metabolic origin, however, 1,4-NQs have key roles in the interactions certain plants have with their biotic environment. New evidence has also emerged demonstrating that menadione, a synthetic 1,4-NQ, which is also present in *Juglans*,^70^ is capable of priming crops against abiotic stress.^295^ This raises the prospect that other plant 1,4-NQ natural products may also have similar roles *in planta*.

Medicinal plants synthesizing 1,4-NQs have been used for centuries based on the numerous pharmacological applications of these compounds.^7^ Today, molecular studies are beginning to validate these claims (see text and Table 2), making 1,4-NQs strong candidates for developing novel drugs (for example, shikonin against breast cancer^296^). The 1,4-NQs are also targets for synthetic strategies to develop more potent therapeutics, though there is undoubtedly a number of plant 1,4-NQs still undiscovered in nature that may already offer such drugs.

Beyond the core metabolic pathways (for example, phenylpropanoid, benzenoid and terpenoid) providing precursors for synthesizing specialized 1,4-NQs, there are only a couple identified 1,4-NQ biosynthetic genes. Although there is solid biochemical support for the involvement of these genes, there is a lack of genetic evidence to corroborate these results. This is in part due to the lack of genetically amenable systems in which to study these pathways. Therefore, there is a critical need to develop methods for generating transgenics in 1,4-NQ-synthesizing species, thus allowing for functional screening of candidates identified, for example, by comparative transcriptomic approaches. With the decreased costs and increased capabilities of sequencing technologies, forward genetic screens in existing horticultural models are also a favorable strategy to consider.

Indications are that biosynthesis of plant 1,4-NQs is highly compartmentalized and spread across multiple subcellular locations. This implicates the contribution of undefined transporters and other protein-mediated trafficking steps to the movement of metabolic intermediates and 1,4-NQs throughout the cell. That plant 1,4-NQs are often secreted into the environment further suggests the involvement of unknown plasma membrane-localized transporters and/or vesicular exocytosis for deployment.^165,297–299^

Despite extensive work over the last several decades, specialized plant 1,4-NQs are clearly an understudied, yet extremely promising class of metabolites for developing novel drugs and innovative strategies to address horticultural challenges, especially in pest management. To harness these metabolites for practical applications, however, will require a monumental improvement in the basic knowledge encompassing 1,4-NQ synthesis, transport and the molecular mechanisms behind their modes of action and release into the environment.

## Figures and Tables

**Table 1:** Major 1,4-NQ natural products produced by horticultural species highlighted in this review

*Common name*	*Scientific name*	*Major 1,4-NQ natural product(s) present*
*Medicinal or ethnobotanical*		
Henna	*Lawsonia inermis*	Lawsone
Pau d’arco tree	*Tabebuia impetiginosa*	Lapachol
Madder	*Rubia tinctorum*	Alizarin
Purple gromwell (Zi cao) Arnebia	*Lithospermum erythrorhizon* *Arnebia euchroma*	Shikonins
Alkanet Arizona popcorn flower	*Alkanna tinctoria* *Plagiobothrys arizonicus*	Alkannins
Pipsissewa One-flowered wintergreen	*Chimaphila umbellate* *Moneses uniflora*	Chimaphilins
Indian leadwort	*Plumbago indica*	Plumbagin
		
*Ornamental*		
Garden balsam	*Impatiens balsamina*	Lawsone
Himalayan balsam	*Impatiens glandulifera*	Lawsone, 2-MNQ
Venus fly trap	*Dionaea muscipula*	Plumbagin, droserone
Pitcher plants	*Nepenthes* sp.	Plumbagin, droserone, 7-Methyljuglone
		
*Nuts and seeds*		
Black walnut English walnut	*Juglans nigra* *Juglans regia*	Juglone
Pecan	*Carya illinoensis*	Juglone
Sesame	*Sesamum indicum*	Anthrasesamones

Abbreviations: 1,4-NQ, 1,4-naphthoquinone; 2-MNQ, 2-methoxy-1,4-NQ.

**Table 2:** Additional examples of pharmacological mechanisms of action for plant-derived 1,4-NQs

*Plant-derived 1,4-NQ(s)*	*Pharmacological mechanism of action*	*Reference(s)*
Shikonin	Protection of brain against ischemic stroke damage by attenuated TLR4, p-p38MAPK, NF-κB, TNF-α and MMP-9 expression, and upregulated claudin-5 expression	^255^
	Suppression of epithelial–mesenchymal transition and downregulation of expression of Slug and MMP-2, -9 and -14 in thyroid cancer cells	^223^
	Management of inflammatory bowel disease by inhibiting activation of NF-κB and STAT3	^256^
	Promotion of intestinal wound healing via induction of TGF-β release	^257^
	Inhibition of expression of the pro-inflammatory cytokine TNF-α through selective blockade of pre-mRNA splicing	^258^
	Inhibition of IFN-γ induced K17 overexpression by interfering with STAT3 signaling in psoriasis pathogenesis	^259^
	Inhibition of lipopolysaccharide-induced release of HMGB1 via IFN-β and NF-κB signaling pathways in inflammation	^260^
	Inhibition of STAT3-, FAK- and Src-mediated signaling in breast cancer	^261^
	Suppression of IL-17-induced VEGF expression via blockage of the JAK2/STAT3 pathway in psoriasis pathogenesis	^262^
	Inhibition of c-MYC expression with involvement of ERK/JNK/MAPK and AKT pathways in leukemia cells	^263^
	Suppression of orphan nuclear receptor Nr4a family gene expression for treating allergic diseases	^264^
Shikonin derivatives	Inhibition of the transcriptional activation of the human TNF-α promoter in treating inflammatory diseases	^265^
	Inhibition of tumor angiogenesis via inhibition of VEGFRs	^266^
Shikonin and alkannin	Inhibition of cancer cell glycolysis via inhibition of tumor-specific PKM2	^267^
Plumbagin	Decreased expression of TNF-α, IFN-γ and IL-17 in murine ulcerative colitis	^268^
	Amelioration of autoimmune encephalomyelitis via downregulation of JAK–STAT and NF-κB signaling pathways	^269^
	Antiproliferative activity against lung epithelium carcinoma cells by disruption of the microtubule network through tubulin binding	^270^
	Inhibition of cytochrome P450s	^271,272^
	Inhibition of telomerase and induction of cell death in human brain tumor cells	^273^
	Induction of cell cycle arrest and autophagy; suppression of epithelial to mesenchymal transition involving the PI3K/Akt/mTOR-mediated pathway in human pancreatic cancer cells	^274^
	Induction of G2/M arrest, apoptosis, and autophagy via p38 MAPK- and PI3K/Akt/mTOR-mediated pathways in human tongue carcinoma cells	^275^
	Binding to and inhibition of five cancer signaling proteins (PI3Kc, AKT1/PKBa, Bcl-2, NF-κB and Stat3)	^276^
	Interference with the binding of ER-alpha to ERE and antagonism at the death receptor complex in BRCA1 breast cancer cells	^277^
5-*O*-Acyl plumbagins	Inhibition of mammalian DNA polymerase and suppression of inflammatory response	^278^
7-Methyljuglone	Suppression of PI3K/Akt signaling in breast cancer cells	^279^
Juglone	Inactivation of cysteine-rich proteins required for progression through mitosis	^251^
	Prevention of metabolic endotoxemia-induced hepatitis and neuroinflammation via suppression of the TLR4/NF-κB signaling pathway	^280^
Juglone and plumbagin	Inhibition of protein tyrosine phosphatases, leading to increased phosphorylation and activation of epidermal growth factor receptor in HaCaT keratinocytes	^219^
Acetylshikonin, shikonin, juglone, lawsone, plumbagin and lapachol	Inhibition of monoamine oxidases regulating neurotransmitter levels and cell signaling, growth and differentiation	^281–283^

Abbreviations: ERE, estrogen responsive elements; IFN, interferon; IL-17, interleukin-17; MMP, matrix metallopeptidase; NF-κB, nuclear factor κB; 1,4-NQ, 1,4-naphthoquinone; PKM2, pyruvate kinase-M2; TGF-β, transforming growth factor-β; TLR4, Toll-like receptor 4; TNF-α, tumor-necrosis factor-α; VEGF, vascular endothelial growth factor; VEGFR, VEGF receptor.
